# Purpurin Triggers Caspase-Independent Apoptosis in *Candida dubliniensis* Biofilms

**DOI:** 10.1371/journal.pone.0086032

**Published:** 2013-12-23

**Authors:** Paul Wai-Kei Tsang, Alan Pak-Kin Wong, Hai-Ping Yang, Ngai-For Li

**Affiliations:** Oral BioSciences, Faculty of Dentistry, the University of Hong Kong, HKSAR, China; Louisiana State University, United States of America

## Abstract

*Candida dubliniensis* is an important human fungal pathogen that causes oral infections in patients with AIDS and diabetes mellitus. However, *C*. *Dubliniensis* has been frequently reported in bloodstream infections in clinical settings. Like its phylogenetically related virulent species C. albicans, *C*. *Dubliniensis* is able to grow and switch between yeast form and filamentous form (hyphae) and develops biofilms on both abiotic and biotic surfaces. Biofilms are recalcitrant to antifungal therapies and *C*. *Dubliniensis* readily turns drug resistant upon repeated exposure. More than 80% of infections are associated with biofilms. Suppression of fungal biofilms may therefore represent a viable antifungal strategy with clinical relevance. Here, we report that C. dubliniensis biofilms were inhibited by purpurin, a natural anthraquinone pigment isolated from madder root. Purpurin inhibited C. dubliniensis biofilm formation in a concentration-dependent manner; while mature biofilms were less susceptible to purpurin. Scanning electron microscopy (SEM) analysis revealed scanty structure consisting of yeast cells in purpurin-treated C. dubliniensis biofilms. We sought to delineate the mechanisms of the anti-biofilm activity of purpurin on *C*. *Dubliniensis*. Intracellular ROS levels were significantly elevated in fungal biofilms and depolarization of MMP was evident upon purpurin treatment in a concentration-dependent manner. DNA degradation was evident. However, no activated metacaspase could be detected. Together, purpurin triggered metacaspase-independent apoptosis in C. dubliniensis biofilms.

## Introduction

Invasive fungal infections have long been one of the most important medical problems in humans [1-3]. *Candida* fungi, among others, are prevalent human fungal pathogens that cause both superficial and systemic diseases (candidiasis) in patients with impaired immunity. In severe cases, the mortality and morbidity range from 40-60% [4,5]. In fact, candidiasis is the fourth leading type of hospital-acquired infections in clinical settings [6,7]. Although C. albicans is the major causative agent of candidiasis, recent epidemiological and clinical studies have shown an escalating number of bloodstream infections caused by non-*albicans Candida* species, which accounted for 36-63% of candidemia [8-11].

*C*. *dubliniensis* is now firmly recognized as an emerging and medically-relevant opportunistic human fungal pathogen, especially in the oral cavity of patients with AIDS and diabetes mellitus. Epidemiological studies indicated a worldwide spread of *C*. *dubliniensis*-related infections [12,13]. *C*. *dubliniensis* has been isolated from other body sites including respiratory tract and blood [14,15], with up to 7% of candidemia caused by this pathogenic fungus [16]. In addition, azole-resistant *C*. *dubliniensis* isolates have been frequently reported in antifungal interventions, in particular to repeated and lengthy treatments [17,18], suggesting a dire need for novel approaches and strategies in treating this notorious human fungal pathogen.

 In the course of our continuing efforts to characterize small molecules with novel antifungal activity, we have demonstrated the potent *in vitro* antifungal activity of purpurin, a natural red anthraquinone pigment in madder roots (*Rubia tinctorum* L.), against a panel of six pathogenic *Candida* species [19]. In particular, purpurin was found inhibitory to C. albicans biofilm development by downregulation of the expression of hypha-specific genes and the central morphogenetic regulator Ras1p [20]. *C*. *dubliniensis* is a close relative to C. albicans as they share >80% identity in genome sequence [21]. *C*. *dubliniensis* and C. albicans are the only *Candida* species that can form true hyphae [22], and C. dubliniensis biofilms have been documented in bloodstream infections and on the surfaces of different biomaterials in the presence of saliva and serum [23-25]. *Candida* biofilms are heterogeneous sessile communities of yeast and hyphal cells in extracellular matrix, and are highly resistant to antifungal chemotherapy [26,27]. It has been estimated that 80% of microbial infections are biofilm-associated [28]. Thus, suppression of biofilms could be an effective measure to tackle with *Candida* virulence and pathobiology. The present study was designed to investigate the *in vitro* effect of purpurin on C. dubliniensis biofilms, with special attention on its correlation to cell demise.

## Results and Discussion

The main etiopathologic role of biofilms in pathogenic *Candida* fungi is its ability to maintain a community of invasive fungal population that serves as a reservoir for recurrent host infection and disease dissemination [29]. Similar to its phylogenetically closely related species C. albicans, *C*. *dubliniensis* forms true hyphae and biofilms on both abiotic and biotic surfaces [24]. The increasing clinical prevalence of *C*. *dubliniensis* as an emerging human fungal pathogen is reflected by an astonishing number of reports in detecting bloodstream isolates and medical devices such as catheters and implanted materials [17,18,30].

**Figure 1:**
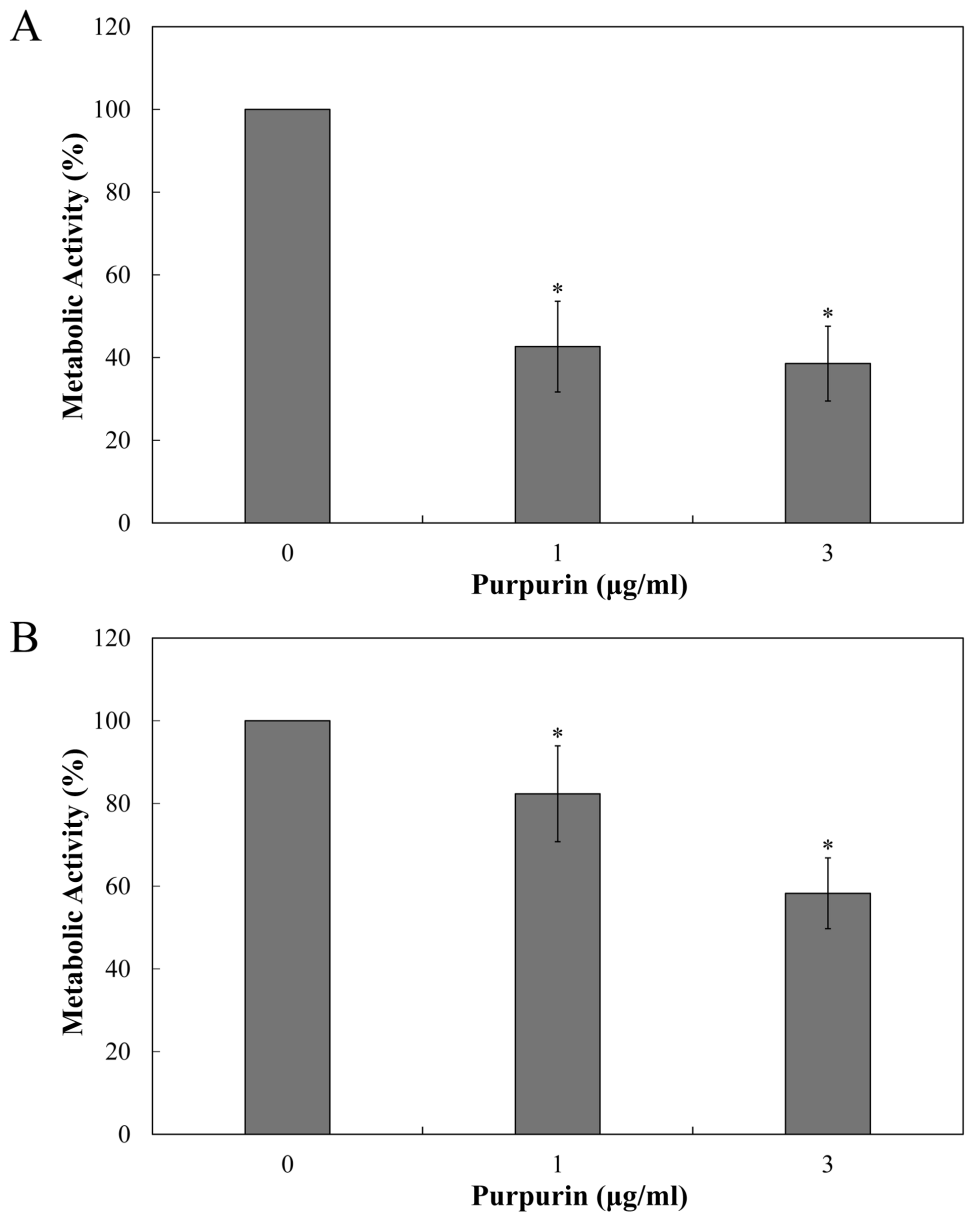
Effect of purpurin on *Candida dubliniensis* biofilms. A) Biofilm formation of C. dubliniensis MYA-646. B) Mature biofilms. In both cases, biofilms were incubated with the indicated concentration of purpurin for 24 h at 37°C. The metabolic activity of the biofilms was assessed semi-quantitatively using XTT reduction assay. The activity of samples without purpurin treatment (i.e. DMSO only) (0 µg/ml) was taken as 100%. Results shown were the average of three independent experiments ± SD. *p<0.05 when compared with the untreated controls.

**Figure 2:**
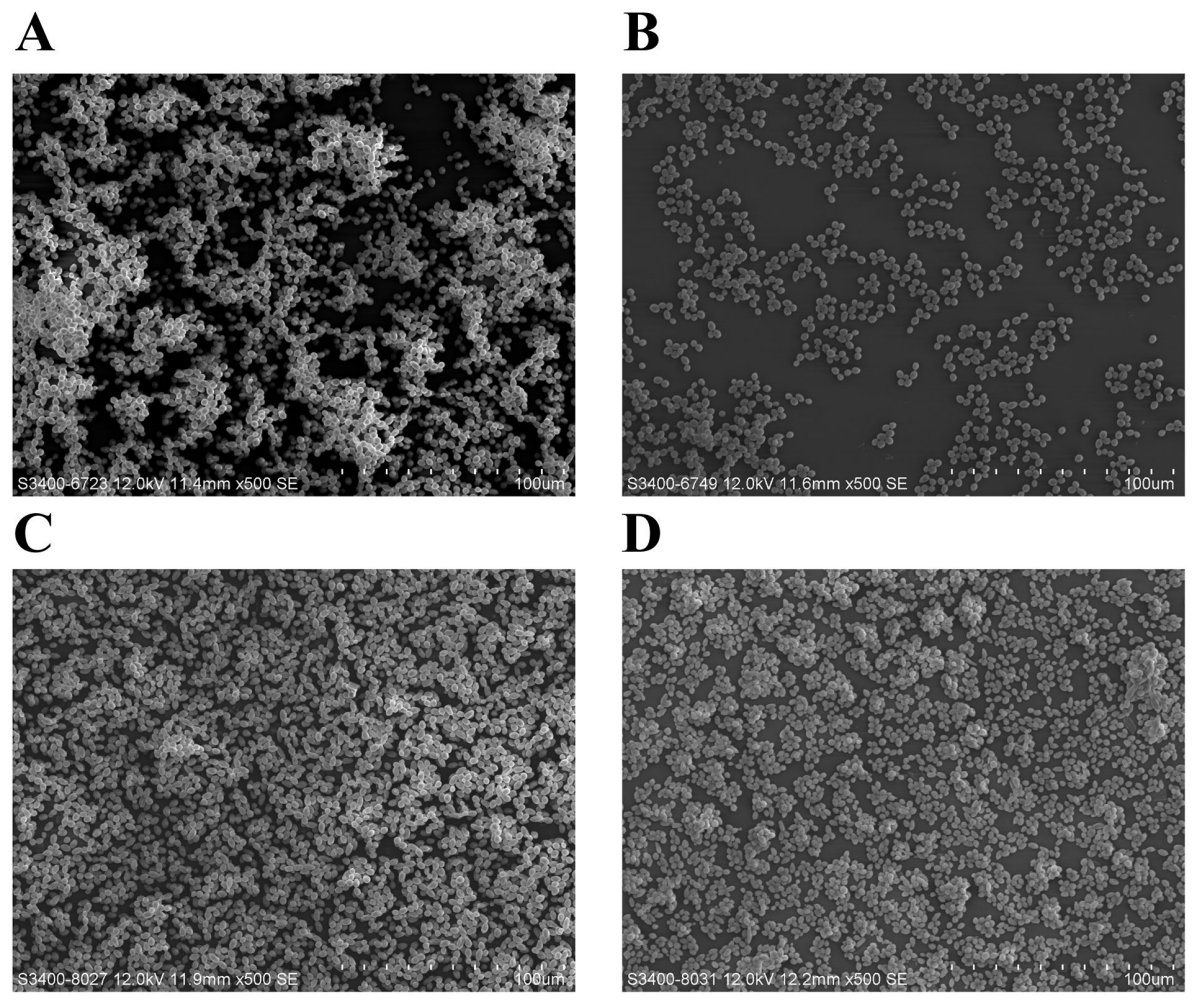
Scanning electron microscopy images of *Candida dubliniensis* biofilms. Representative SEM images showing the topography of C. dubliniensis biofilms. Fungal biofilms were formed on plastic coverslips, processed, and coated with gold before viewing (magnification 500×). A) Biofilm formation in the absence of purpurin. B) Biofilm formation in the presence of purpurin (1 µg/ml). C) Pre-formed biofilms in the absence of purpurin. D) Pre-formed biofilms in the presence of purpurin (1 µg/ml).

Our laboratory recently deciphered the mechanisms of action of purpurin against *Candida* fungi through perturbation of mitochondrial homeostasis and initiation of apoptosis [19]. More experiments indicated that purpurin also interferes with C. albicans biofilm development at sub-MIC levels (3 µg/ml) [20]. In the present study, we explored the antifungal activity of purpurin on C. dubliniensis biofilms and evaluated several biochemical hallmarks of cell demise (apoptosis), an area that is still relatively unexplored in this *Candida* species. The antifungal activity of purpurin on C. dubliniensis biofilms was evaluated semi-quantitatively by using 3-bis(2-methoxy-4-nitro-5-sulfo-phenyl)-2H-tetrazolium-5-carboxanilide (XTT) reduction assay. The inhibitory effect was concentration-dependent, as revealed by a progressive reduction in cell viability with increasing concentrations of purpurin (Figure 1A). The metabolic activity of *C*. *dubliniensis* during biofilm formation was reduced by ~45% at 1 µg/ml and by ~65% at 3 µg/ml. Pre-formed (mature) C. dubliniensis biofilms were less susceptible to purpurin; the cell viability was reduced by ~17% at 1 µg/ml and by ~40% at 3 µg/ml (Figure 1B). Scanning electron microscopy (SEM) images showed that purpurin inhibited biofilm development in *C*. *dubliniensis*, a scanty architecture with reduced hyphal growth was evident in purpurin-treated biofilms (Figure 2A-D). Considering the differences in phenotypic properties of *C*. *dubliniensis* to its sibling C. albicans, our data demonstrated that *C*. *dubliniensis* is more vulnerable as lower concentration of purpurin (1 µg/ml) was sufficient to abrogate biofilm development. As in other biofilm-forming microbes, the structurally well-organized mature C. dubliniensis biofilms were more resistant to purpurin, presumably due to poor penetration.

 The *in vitro* effect of purpurin on C. dubliniensis biofilms prompted us to evaluate the presence of apoptotic features, namely reactive oxygen species (ROS), mitochondrial membrane potential (MMP), and DNA degradation, upon exposure to purpurin. Apoptosis is a highly sophisticated cellular process that leads to cell death in multicellular organisms and is crucial for normal growth, development, and cell maintenance. *Candida* fungi exhibit biochemical hallmarks typical to mammalian apoptosis in response to diverse environmental cues [31-36], in which mitochondria play a pivotal role in controlling both caspase-dependent and caspase-independent cell death pathways. Mitochondrial dysfunction is regarded as the onset of apoptosis [37-40]. Moreover, it has been shown that activation of C. albicans metacaspase is associated with elevation of intracellular ROS levels [41].

**Figure 3:**
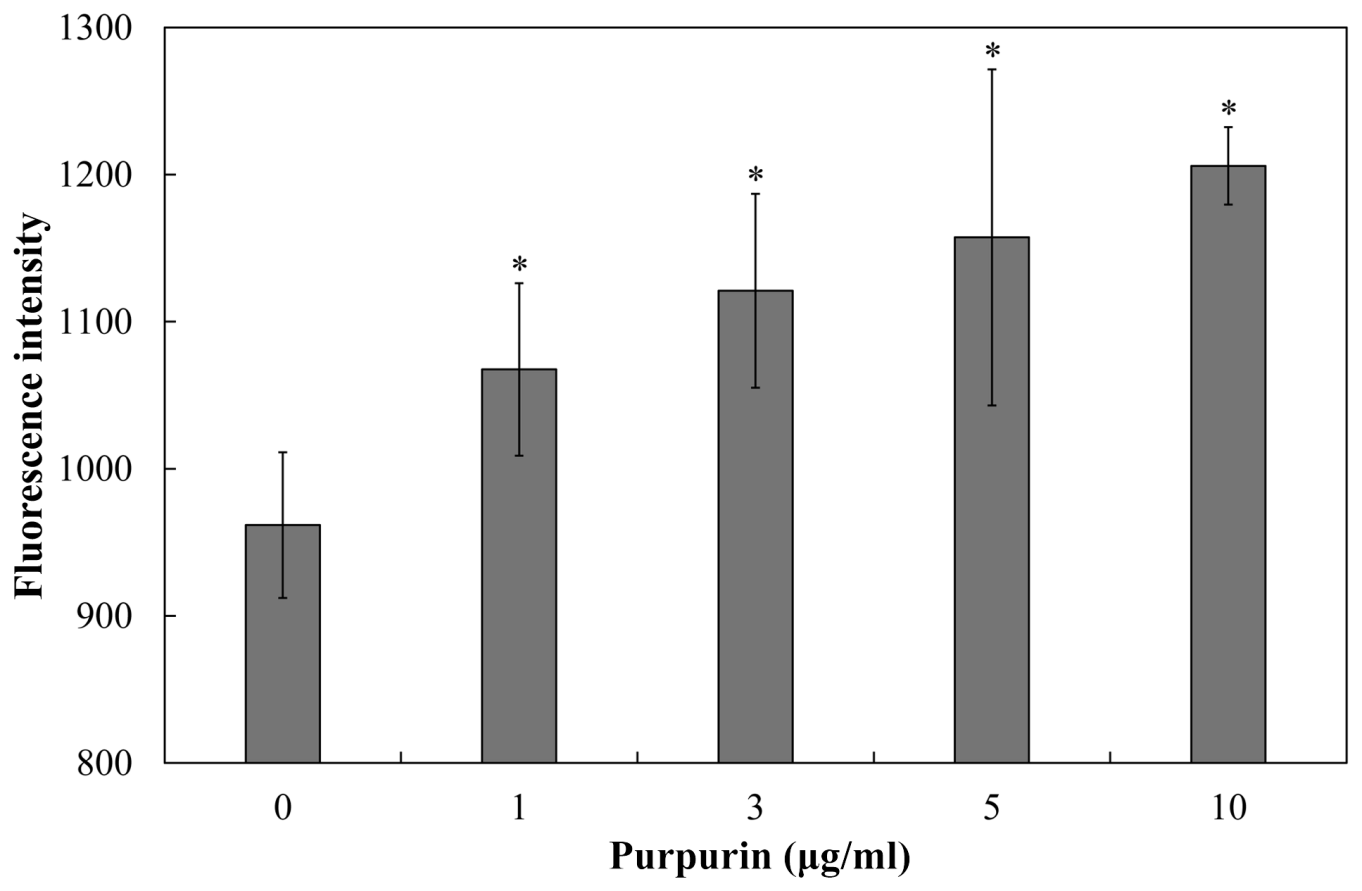
Effect of purpurin on intracellular ROS levels. C. dubliniensis biofilms were treated with different concentrations of purpurin (from 1 µg/ml to 10 µg/ml), and intracellular ROS levels were determined using DCFDA. Fluorescence intensity was measured at 485 nm excitation and 535 nm emission. Results shown were the average of three independent experiments ± SD. *p<0.05 when compared with the untreated controls.

**Figure 4:**
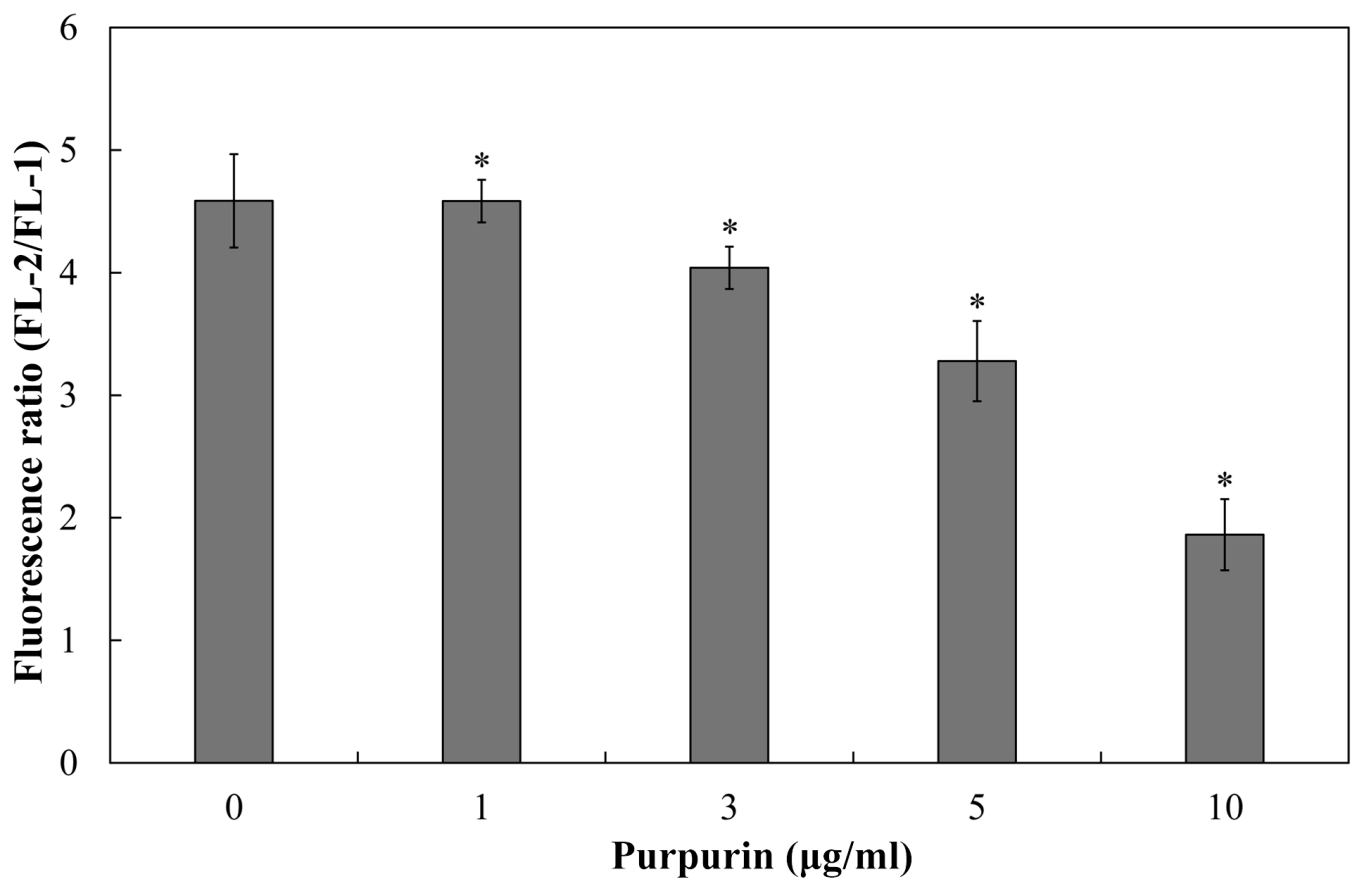
Depolarization of MMP in purpurin-treated *Candida dubliniensis* biofilms. Purpurin-treated C. dubliniensis biofilms were incubated with JC-1. MMP was measured by flow cytometer at FL-1 (525 nm) and FL-2 (595 nm), and expressed as ratio of FL-2/FL-1. Results shown were the average of three independent experiments ± SD. *p<0.05 when compared with the untreated controls.

Therefore, we sought to evaluate the effect of purpurin on mitochondrial homeostasis. Purpurin increased the intracellular ROS levels in C. dubliniensis biofilms in a concentration-dependent manner, as revealed by a progressive elevation of fluorescence intensity. At 1 µg/ml, the endogenous ROS levels were increased by ~11%, and by ~27% at higher concentration (10 µg/ml) (Figure 3). Immense elevation of intracellular ROS levels is regarded as an onset of apoptosis, which is followed by depolarization of MMP [42]. To this end, we used the lipophilic fluorescent dye 5,5’,6,6’-tetrachloro-1,1’,3,3’-tetraethyl-imidacarbocyanine iodide (JC-1) to measure MMP in purpurin-treated C. dubliniensis biofilms. Intact MMP allows the cationic dye to enter the mitochondrial matrix and form J-aggregates (red fluorescence) beyond critical concentration. On the contrary, cells with depolarized MMP fluoresce green due to monomeric JC-1 [19]. The ratio of red fluorescence (FL-2) to green fluorescence (FL-1) represents the change of MMP in cell population. As expected, purpurin elicited a concentration-dependent depolarization of MMP in C. dubliniensis biofilms. At 1 µg/ml, MMP was decreased by ~12% and by 57 % at 10 µg/ml (Figure 4).

**Figure 5:**
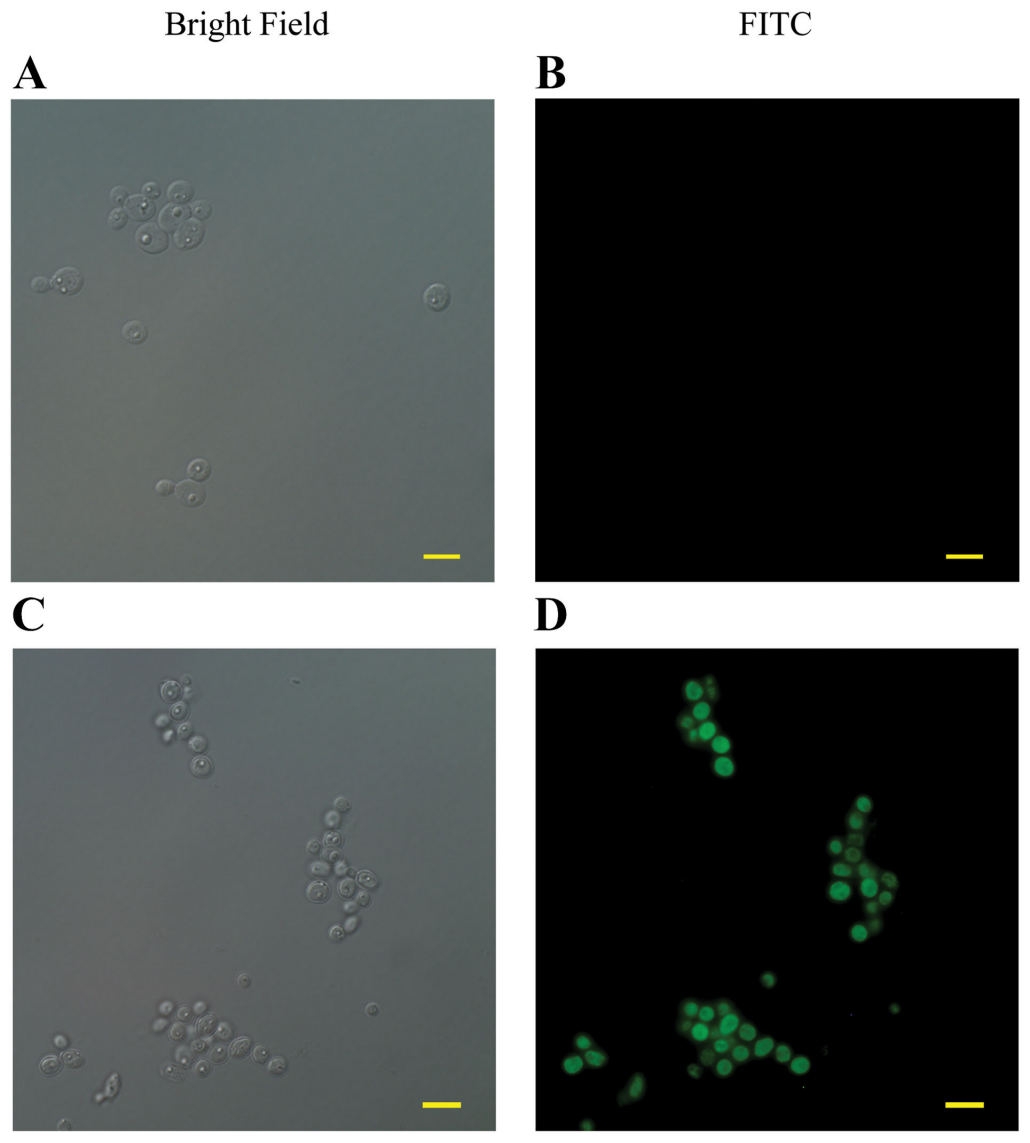
Effect of purpurin on DNA integrity (TUNEL assay). Representative fluorescent images showing the occurrence of DNA degradation in purpurin-treated C. dubliniensis biofilms captured by confocal microscope. A) Biofilms in the absence of purpurin (Bright Field). B) Biofilms in the absence of purpurin (FITC). C) Biofilms in the presence of purpurin (1 μg/ml) (Bright Field). D) Biofilms in the presence of purpurin (1 μg/ml) (FITC). Cells with damaged DNA emitted intense green fluorescence. The bar represents 50 µm.

Collapse of MMP triggers a series of downstream cellular events that lead to disorganization of DNA and therefore cell death [43,44]. We assessed the effect of purpurin on DNA integrity in C. dubliniensis biofilms by using the TUNEL assay. DNA degradation results in a multitude of 3’-OH termini of DNA ends that can be labelled by fluorescent-tagged deoxyuridine triphosphate nucleotides using terminal deoxynucleotidyl transferase-mediated dUTP nick end labelling (TUNEL) assay [45]. Untreated fungal cells did not emit green fluorescence; while TUNEL-positive cells (green fluorescence) were detected upon purpurin treatment in C. dubliniensis biofilms (Figure 5A-D).

Activation of caspase is a distinct biochemical marker of apoptosis. Although no *bona fide* caspase is found in fungi, a distant relative called metacaspase has been characterized in *Saccharomyces cerevisiae* and C. albicans [41,46]. We therefore examined the presence of activated metacaspases in purpurin-treated C. dubliniensis biofilms by using FITC-VAD-FMK. FITC-VAD-FMK is a cell permeable caspase inhibitor that specifically binds to activated caspases [47]. However, no green fluorescence could be observed in purpurin-treated C. dubliniensis biofilms (data not shown), suggesting an absence of activated metacaspases. Nevertheless, a putative metacaspase sequence (Cd36_85170) has been annotated in *C*. *dubliniensis* genome (http://www.candidagenome.org/), and we have detected the presence of activated metacaspase upon exposure of planktonic cells of *C*. *dubliniensis* to apoptotic levels of amphotericin B or acetic acid (P. W. K. Tsang, unpublished data). Functional significance of this uncharacterized open reading frame in apoptosis is in progress in our laboratory.

*C*. *dubliniensis* poses a serious health threat to humans and puts heavy economic burden on our society. Strategies that work by switching on endogenous cell death mechanisms specific to *C*. *dubliniensis* could be a novel and tangible antifungal approach against this emerging human fungal pathogen. Our findings clearly demonstrated that purpurin triggers mitochondrial-mediated apoptotic pathway in C. dubliniensis biofilms without the involvement of activated metacaspases, suggesting that purpurin-mediated cell death in *C*. *dubliniensis* does not require the proteolytic activity of metacaspases. This is an entirely new antifungal action mechanism which is different from the standard antifungal agents in clinical settings: flucytosine inhibits DNA synthesis; polyenes cause membrane damage; azoles affect sterol metabolism; and echinocandins inhibit cell wall synthesis. Nevertheless, the usefulness of these antifungal agents has been hampered by severe side effects, poor therapeutic index, and the emergence of multidrug resistance [48,49]. Therefore, the unique antifungal action of purpurin on *C*. *dubliniensis* could be of particular clinical relevance in the design of more selective and effective therapeutic treatments. One aspect could be killing of C. dubliniensis biofilms via induction of the endogenous mitochondrial-mediated apoptotic pathway. Another aspect could be a combined use of purpurin with the conventional antifungal agents to reduce potential side effects and the likelihood of acquired multidrug resistance.

In conclusion, the findings of the present study provide solid evidence that purpurin triggered apoptosis-like features in C. dubliniensis biofilms. As *C*. *dubliniensis* and C. albicans belong to the CTG clade, it is not surprising that they share common core pathways in metabolism. Nonetheless, a comparative analysis of calcineurin signalling pathways between *C*. *dubliniensis* and C. albicans indicated differential scenarios in pH homeostasis [50]. Another study revealed a rewiring of iron assimilation gene expression in *C*. *dubliniensis* [51]. Thus, comparing metabolic pathways between these two species not only helps gain further insights into their evolutionary divergence, but also alludes to a functional characterization of core machineries responsible for their pathogenicity. For instance, a better understanding of the cell death mechanisms can be beneficial to the design of innovative antifungal strategies that target the core components specifically.

## Materials and Methods

### Strains, cultivation and chemicals

The reference laboratory strain *C*. *dubliniensis* MYA-646 was used throughout the study, and was routinely cultured in YPD agar (10 g/l yeast extract, 20 g/l peptone, 20 g/l dextrose, 20 g/l agar) at 30°C. To prepare a standard cell suspension, a single colony was inoculated into YNB medium (6.7 g/l yeast nitrogen base w/o amino acids, 20 g/l dextrose) and incubated for 18 h at 30°C with agitation (200 rpm). Fungal cells were harvested by centrifugation, washed twice with phosphate-buffered saline (PBS) (pH 7.2), and resuspended at 1 × 10^7^ cells/ml. Purpurin (purity >99%) was purchased from TimTec Inc. (Newark, DE, USA). Stock solution of purpurin was prepared in distilled dimethyl sulphoxide (DMSO) and kept at -20°C until use. The final concentration of DMSO was 1% in all assays. Other chemicals were obtained from commercial suppliers with the highest grade available.

### Effect of Purpurin on C. Dubliniensis Biofilm Formation and Pre-Formed (Mature) Biofilms

Fungal biofilms were prepared as described [52] on commercially available, pre-sterilized, flat-bottomed 96-well polystyrene microtitre plates (Iwaki). Standard cell suspension of *C*. *dubliniensis* (100 µl) was transferred into the wells and incubated for 1.5 h at 37°C with agitation (80 rpm). After the adhesion phase, the liquid was aspirated and the wells were washed twice with PBS to remove non-adherent cells. Fresh YNB medium (200 µl) containing purpurin (1 µg/ml or 3 µg/ml) was added to each well and the plates were incubated for an additional 24 h at 37°C.

To investigate the effect of purpurin on pre-formed biofilms, C. dubliniensis biofilms were prepared for 24 h at 37°C as described above. The wells were washed twice with PBS and fresh YNB medium (200 µl) containing purpurin (1 µg/ml or 3 µg/ml) was added and the plates were incubated for an additional 24 h at 37°C. YNB medium with 1% DMSO was included in control wells. The metabolic activity of the C. dubliniensis biofilms, representing viability of the fungal cells, was determined by using a standard XTT reduction assay that measures mitochondrial dehydrogenase activity [52]. XTT solution (1 g/l; 40 µl) was mixed with freshly prepared menadione solution (0.4 mM; 2 μl) at 20:1 (v/v) immediately prior to the assay. Thereafter, PBS (158 µl) was mixed with XTT-menadione solution (42 µl) and transferred to each well containing pre-washed biofilms, and incubated in the dark for 3 h at 37°C. Colour changes were measured at 492 nm with a microtitre plate reader (SpectraMax 340 tunable microplate reader; Molecular Devices).

### Measurement of endogenous ROS levels

The effect of purpurin on intracellular ROS levels in C. dubliniensis biofilms was examined by using the fluorescent dye 2’,7’-dichlorofluorescein diacetate (DCFDA) (Molecular Probes, CA, USA) [45]. Briefly, after treatment with different concentrations of purpurin (from 1 µg/ml to 10 µg/ml), the biofilms were washed twice with PBS, added with DCFDA (final concentration: 20 µM) and incubated for 1 h at 37°C. Fluorescence intensity was measured in a fluorescence plate reader (TECAN Polarion, Tecan UK Ltd, Theale, UK) with a 485 nm excitation and 535 nm emission.

### Measurement of MMP

The effect of purpurin on MMP of C. dubliniensis biofilms was analyzed as described by using JC-1 [19]. Briefly, after treatment with different concentrations of purpurin (from 1 µg/ml to 10 µg/ml), the biofilms were washed thrice with PBS, followed by incubation with JC-1 (0.25 µM) (Molecular Probes, CA) for 15 min at 35°C. Fluorescence intensities at FL-1 (unhealthy cells emit green fluorescence, 525 nm) and FL-2 (healthy cells emit red fluorescence, 595 nm) were recorded by a Beckman-Coulter flow cytometer, and the results were expressed as a ratio of the mean values at FL-2 and FL-1.

### Detection of activated metacaspases

The presence of activated metacaspases in C. dubliniensis biofilms was evaluated by using CaspACE™ FITC-VAD-FMK In Situ Marker (Promega, Madison, WI, USA). Briefly, purpurin-treated C. dubliniensis biofilms were washed thrice with PBS and incubated with CaspACE™ FITC-VAD-FMK In Situ Marker (10 µM) for 20 min at 37°C in the dark. The cells were washed twice and resuspended in PBS. Aliquots of fungal cells were added to poly-L-lysine-coated glass slides and analyzed by fluorescence microscopy using a confocal laser scanning microscope (Fluoview FV 1000, Olympus, Tokyo, Japan). Fungal cells containing activated metacaspases fluoresced green.

### TUNEL assay

DNA fragmentation in C. dubliniensis biofilms after treating with purpurin was detected by using the *In Situ* Cell Death Detection Kit (fluorescein) (Roche, Penzbery, Germany) [31]. Briefly, after incubation with purpurin, the biofilms were washed thrice with PBS and fixed with 2% paraformaldehyde for 1 h at 20°C. The biofilms were rinsed thrice with PBS, and then incubated with permeabilization solution (0.1% Triton X-100, 0.1% sodium citrate) on ice for 2 min. The biofilms were rinsed twice with PBS and labelled with 50 µl TUNEL reaction mixture for 1 h at 37°C in a humidified incubator in the dark. The cells were mounted under glass coverslip and analyzed by fluorescence microscopy using a confocal laser scanning microscope.

### SEM analysis

C. dubliniensis biofilms were prepared on custom-made, tissue culture-treated, polystyrene coverslips as described [20,52]. Thereafter, the coverslips were washed twice with PBS and placed in 1% osmium tetroxide for 1 h. Samples were subsequently washed with distilled water, dehydrated in a series of ethanol solutions (70% for 10 min, 95% for 10 min and 100% for 20 min), and air-dried overnight in a desiccator prior to sputter coating with gold (JFC1 100; JEOL). The surface topographies of the C. dubliniensis biofilms were viewed with a scanning electron microscope (Philip XL30CP).

### Statistical analysis

All experiments were performed in triplicate in three different occasions and all data were expressed as the mean values with the corresponding standard deviation (SD). Statistical significance between treated and control groups was assessed by Mann-Whitney U test. A *p*-value of <0.05 was considered statistically significant.
